# A Tractography-Based Grading Scale of Brain Arteriovenous Malformations Close to the Corticospinal Tract to Predict Motor Outcome After Surgery

**DOI:** 10.3389/fneur.2019.00761

**Published:** 2019-07-17

**Authors:** Maogui Li, Pengjun Jiang, Rui Guo, Qingyuan Liu, Shuzhe Yang, Jun Wu, Yong Cao, Shuo Wang

**Affiliations:** ^1^Department of Neurosurgery, Beijing Tiantan Hospital, Capital Medical University, Beijing, China; ^2^China National Clinical Research Center for Neurological Diseases, Beijing, China; ^3^Center of Stroke, Beijing Institute for Brain Disorders, Beijing, China; ^4^Beijing Key Laboratory of Translational Medicine for Cerebrovascular Diseases, Beijing, China

**Keywords:** arteriovenous malformation, corticospinal tract, patient selection, surgery, tractography

## Abstract

**Background:** Surgical decision-making for brain arteriovenous malformations (AVMs) close to the corticospinal tract (CST) is always challenging. The purpose of this study was to develop a tractography-based grading scale to improve preoperative risk prediction and patient selection.

**Methods:** We analyzed a consecutive, surgically treated series of 90 patients with AVMs within a 10-mm range from the CST demonstrated by preoperative diffusion tensor tractography. Poor motor outcome was defined as persistent postoperative limb weakness. We examined the predictive ability of nidus-to-CST distance (NCD), the closest CST level (CCL), deep perforating artery supply, as well as variables of the supplemented Spetzler-Martin grading system. Three logistic models were derived from different multivariable logistic regression analyses, of which the most predictive model was selected to construct a prediction grading scale. Receiver operating characteristic analysis was conducted to test the predictive accuracy of the grading scale.

**Results:** Twenty-one (23.3%) patients experienced persistent postoperative limb weakness after a mean 2.7-year follow-up. The most predictive logistic model showed NCD (*P* = 0.001), CCL (*P* = 0.017), patient age (*P* = 0.004), and AVM diffuseness (*P* = 0.021) were independent predictors for poor motor outcome. We constructed the CLAD grading scale incorporating these predictors. The predictive accuracy of the CLAD grade was better compared with the supplemented Spetzler-Martin grade (area under curve = 0.84 vs. 0.68, *P* = 0.023).

**Conclusions:** Both NCD and CCL predict motor outcome after resection of AVMs close to the CST. We propose the CLAD grading scale as an effective risk-prediction tool in surgical decision-making.

**Clinical Trial Registration:** www.ClinicalTrials.gov, identifier: NCT01758211 and NCT02868008

## Introduction

Complete resection is the gold standard for treatment of brain arteriovenous malformations (AVMs) to eliminate bleeding risk, but the stakes are frequently high (1). Evolving AVM grading systems have been developed to improve preoperative risk prediction and patient selection (2, 3). Because of the serious influence on quality of life, the increased risk of iatrogenic motor dysfunction is an unavoidable concern in surgical decision-making regarding the nidus close to motor structure. Although AVMs abutting the motor cortex have been widely studied with functional neuroimaging or intraoperative electrical mapping (4–6), less attention was paid to the nidus close to the motor pathways. The corticospinal tract (CST) is the direct pathway of voluntary movements. By using diffusion tensor tractography (DTT), a 3D reconstruction of the CST can be obtained. Therefore, the spatial relationships between the nidus, and CST fibers can be accurately assessed (7, 8). Previously, both the quantity and the level of CST involvement have been evidenced to be good predictors for motor outcome in stroke patients (9, 10). Similarly, the usefulness of CST tractography has been well-described in brain tumor studies (11, 12). However, the literature regarding the use of tractography in AVM patients was mostly limited to a few case reports or small series (8, 13–15). Patient selection for surgery of AVMs located close to the CST remains challenging.

In this tractography-based study, we evaluated whether the closeness of the nidus to the CST as well as the susceptible level of the CST could both predict postoperative motor outcome. We hypothesized that a new grading scale incorporating tractography-based predictors would improve patient selection for microsurgical resection of AVMs close to the CST.

## Materials and Methods

### Study Population

The protocol was approved by the institutional review board of Beijing Tiantan Hospital, Capital Medical University. All subjects gave written informed consent in accordance with the Declaration of Helsinki. Between January 2013 and June 2018, 82 patients with the shortest distance between AVMs and a CST <10 mm were extracted from a prospective cohort of two consecutive clinical trials (www.ClinicalTrials.gov No. NCT01758211 and NCT02868008). Eight patients ineligible to the age criteria (12–60 years) of the trials were additionally involved. At the authors' institution, patients who experienced previous hemorrhage, intractable epilepsy, serious secondary headache, or deteriorating neurological status were considered for AVM resection. Surgery was commonly not recommended for patients with no symptoms or with AVMs of Spetzler-Martin (S-M) grade 5. AVM resections were done by two experienced neurosurgeons (YC and SW).

### Imaging Protocol

All patients had undergone magnetic resonance imaging (MRI) and digital subtraction angiography (DSA) examinations within 2 weeks before AVM surgery. Routine four-vessel DSA was performed to evaluate the angioarchitecture of AVMs. DTI was performed combined with 3D anatomical T1-weighted images (T1WI) and 3D time-of-flight MR angiography (TOF MRA), by using a 3.0T MR system (Magnetom Trio, Siemens Healthineers, Erlangen, Germany). The anatomical images were obtained in sagittal orientation with magnetization-prepared rapid gradient-echo (MPRAGE) sequences (TR 2,300 ms, TE 2.98 ms, 176 slices, slice thickness 1 mm, flip angle 8°, FOV 256 mm, matrix 64 × 64, voxel size 1 × 1 × 1 mm). The 3D TOF MRA was performed in transverse orientation with TR 22 ms, TE 3.86 ms, 144 slices, slice thickness 1 mm, FOV 220 × 220 mm, and a matrix of 512 × 512. The DTI was acquired with single-shot echo-planar imaging (EPI) sequences with b-value of 0 and 1,000 along 30 diffusion directions (b TR 6,800 ms, TE 93 ms, 50 slices, slice thickness 2.8 mm, FOV 230 × 230 mm, matrix 128 × 128, double averaging). The parallel acquisition technique, generalized autocalibrating partially parallel acquisitions (GRAPPA), was used with an acceleration factor of 2 to shorten the echo train length. Patients were asked to avoid moving or swallowing during scanning. MRI data were transferred to a workstation of iPlan 3.0 (Brainlab AG, Feldkirchen, Germany) for data processing. The sets of DTI and TOF MRA were both fused with the anatomical T1WI sets by automatic 3D rigid registration.

### Data Collection

Patient demographics collected included age and sex. We reviewed preoperative DSA and MRI of each patient to evaluate AVM characteristics. Nidus size (diameter in cm), pattern of venous drainage, and eloquence were determined conventionally according to the S-M grading system (16). Due to the study purpose, the eloquence here referred to motor-eloquent regions, including precentral cortex, internal capsule, and cerebral peduncles. Rupture history was determined from evidence of hemorrhage provided by MRI or computed tomography, regardless of signs or symptoms. The definition of deep perforating artery supply (DPAS) was in accordance with a previous study (17). AVM with diffuse borders on DSA, or with intervening brain parenchyma within the nidus on MRI, was determined to be diffuse.

Patient neurological status at admission, at 7 days after surgery, and at the last telephone follow-up was evaluated separately. The Medical Research Council Scale (MRCS) of 0–5 was used to rank the motor strength. Iatrogenic limb weakness was defined as a postoperative decreased MRCS score of the contralateral limbs. Poor outcome was defined as persistent limb weakness observed at the last follow-up.

### DTT Evaluation

DTT was based on fiber assignment by continuous tracking (FACT) method (18), and was completed by using the tractography software iPlan 3.0 (Brainlab AG, Feldkirchen, Germany). Eddy current distortions were corrected before fiber tracking. A fractional anisotropy (FA) of 0.2 and a minimum fiber length of 70 mm were selected as the thresholds for tracking the CST. The subcortical white matter of the precentral gyrus was selected as the first region of interest (ROI), and the second ROI was placed at the homolateral inferior part of pons. Specific ROIs were set at the corpus callosum, thalamus, or cerebellar peduncles to remove unrelated fibers going to the contralateral hemisphere, thalamus, or cerebellum. The tractography of each patient was done by two experienced investigators with consensus (ML and RG).

We viewed the MRA fused with reconstructed CST slice by slice on sagittal, coronal, and axial planes, the closeness of AVM to the CST was measured as the shortest linear distance between the margins of the nidus, and the most external borders of the CST fibers (nidus-to-CST distance, NCD). An NCD of 0 mm meant direct involvement. According to neuroanatomy atlases, the tracked CST was divided into five parts: cortex, centrum semiovale (CS), corona radiate (CR), posterior limb of the internal capsule (PLIC), and cerebral peduncles (CP). The susceptible level of the CST was determined by which part the closest CST point was located at (the closest CST level, CCL). The CST attenuated when passing downward through PLIC, and therefore the CCL of each patient was dichotomized into two groups: upper CCL group (cortex, CS, and CR) and lower CCL group (PLIC and CP). The evaluations were done by two of the authors (JW and PJ) blinded to patient clinical course.

### Statistical Analysis

Data were statistically analyzed by using Stata/IC 15.1 software (StataCorp, College Station, TX). Continuous variables are presented as mean ± standard deviation (SD), and categorical variables as number (%). We used Chi-square test to compare categorical variables. Continuous variables were compared by Mann-Whitney *U*-test. The NCD, CCL, as well as patient and AVM characteristics were compared between patients with and without persistent limb weakness. Multivariable logistic regression analysis with backward stepwise selection was used to test the correlation of combined variables with motor outcome. The level of significance was set at *P* < 0.05, and all tests were 2-sided.

### Grading Scale Construction

Logistic models were derived from four different multivariable logistic regression analyses (**Table 2**). The first analysis only included the variables of the supplemented S-M (supp S-M) Grading System into stepwise regression. The second included NCD, CCL, and DPAS, combined with the variables of the supp S-M Grading System into regression. The third included the variables with *P*-value < 0.20 in univariable analysis into regression. The fourth only included NCD and CCL into regression. Logtime (the logarithm of follow-up time) was additionally included in each analysis to test the possible influence of the varying follow-up duration. We used receiver operating characteristic (ROC) analysis to test the predictive accuracy of the generated logistic models. The area under each ROC curve (AUC) was compared by using the χ^2^ test. An AUC of 0.70 or more is considered to have good predictive accuracy.

The logistic model with the largest AUC was used to construct the prediction grading scale. The β coefficients were used to weight the values of each predictor. ROC analysis was conducted to compare the AUC between the new grading scale and the supp S-M grading system. Because the scale was generated from the present data sets, a 5-fold cross-validation was conducted to avoid overstating its predictive ability.

## Results

### Patient Demographics and Motor Outcome

Ninety AVM patients with NCD <10 mm were analyzed. Of them, 49 (54.4%) were male, and the mean age was 27.2 ± 13.1 years. Nine (10.0%) patients presented with impaired motor function at admission. Contralateral limb weakness was observed in 41 (45.6%) patients at 7 days after AVM resection. The follow-up duration ranged from 6 to 69 months with an average of 2.7 ± 1.5 years. No patient died, and 21 (23.3%) patients experienced persistent limb weakness until the last follow-up.

### AVM Characteristics and DTT Findings

Forty-two (46.7%) AVM niduses were ruptured, and 48 (53.3%) were unruptured. By S-M grade, 25 (27.8%) niduses were grade I/II, 50 (55.6%) were grade III, and 15 (16.7%) were grade IV/V. The involvement of conventional motor eloquence was found in 47 (52.2%) niduses. Thirty-one (34.4%) niduses were considered to be diffuse. By DTT evaluation, CST fibers with intact length were reconstructed in 89 patients, and in one patient the corticofugal fibers were found to be disrupted at the corona radiate level because of past AVM rupture. The mean NCD of this series was 4.1 ± 3.4 mm; the CST was directly involved by 27 (30.0%) niduses (NCD = 0 mm). The CCL was higher in 59 (65.6%) niduses, and lower in 31 niduses (34.4%).

### Univariable Analysis

Table 1 compared the patient and AVM variables between patients with and without long-term postoperative limb weakness. Older age (*P* = 0.005), DPAS (*P* = 0.002), shorter NCD (*P* = 0.003), and lower CCL (*P* = 0.048) were significantly associated with poor motor outcome. However, no significant difference was found in unruptured history (*P* = 0.272), size (*P* = 0.192), deep venous drainage (*P* = 0.703), motor eloquence (*P* = 0.130), and diffuseness (*P* = 0.147).

**Table 1:** Comparison of variables between patients with and without persistent postoperative limb weakness^a^.

	**Persistent postop limb weakness**		
**Variable**^b^****	**No**	**Yes**	***P*-value**
Total patients	69 (76.7)	21 (23.3)	
Age, y	25.1 ± 12.2	34.1 ± 13.8	0.005
Sex			0.777
Male	37 (53.6)	12 (57.1)	
Female	32 (46.4)	9 (42.9)	
Preop limb weakness			0.431
No	63 (91.3)	18 (85.7)	
Yes	6 (8.7)	3 (14.3)	
Unrupture history			0.272
No	30 (43.5)	12 (57.1)	
Yes	39 (56.5)	9 (42.9)	
Size, cm	3.7 ± 1.3	4.2 ± 1.5	0.192
Deep venous drainage			0.703
No	49 (71.0)	14 (66.7)	
Yes	20 (29.0)	7 (33.3)	
Motor eloquence			0.130
No	36 (52.2)	7 (33.3)	
Yes	33 (47.8)	14 (66.7)	
Diffuseness			0.147
Compact	48 (69.6)	11 (52.4)	
Diffuse	21 (30.4)	10 (47.6)	
DPAS			0.002
No	46 (66.7)	6 (28.6)	
Yes	23 (33.3)	15 (71.4)	
NCD, mm	4.7 ± 3.4	2.2 ± 2.7	0.003
CCL			0.048
Upper	49 (71.0)	10 (47.6)	
Lower	20 (29.0)	11 (52.4)	
Follow-up, y	2.8 ± 1.5	2.5 ± 1.6	0.451

a *DPAS indicates deep perforating artery supply; NCD, nidus-to-CST distance; CCL, the closest CST level; and CST, corticospinal tract*.

b *Categorical variables are presented as number (%), and continuous variables are presented as mean ± standard deviation*.

### Logistic Regression and ROC Analysis

We obtained three different logistic models from the four logistic regression analyses (Table 2). The logistic model A, consisting of patient age (*P* = 0.002), AVM diffuseness (*P* = 0.048), and motor eloquence (*P* = 0.080), was derived from the first analysis. Age (*P* = 0.004), diffuseness (*P* = 0.021), NCD (*P* = 0.001), and CCL (*P* = 0.017), which were determined as independent predictors by the second as well as the third regression consistently, constituted the logistic model B. The model C involving NCD (*P* = 0.001) and CCL (*P* = 0.005) was derived from the fourth analysis.

**Table 2:** Four Separated Multivariable Logistic Regression Analyses^a^.

	**The first**		**The second**		**The third**		**The fourth**	
**Variable^**b**^**	**OR (95% CI)**	***P*-value**	**OR (95% CI)**	***P*-value**	**OR (95% CI)**	***P*-value**	**OR (95% CI)**	***P*-value**
Size, cm		0.166		0.121		0.121		
Deep venous drainage		0.377		0.675				
Unrupture history		0.467		0.691				
Motor eloquence	2.75 (0.89–8.56)	0.080		0.871		0.871		
Age, per 10 y	2.03 (1.30–3.15)	0.002	2.16 (1.27–3.66)	0.004	2.16 (1.27–3.66)	0.004		
Diffuseness	3.20 (1.01–10.16)	0.048	4.78 (1.26–18.11)	0.021	4.78 (1.26–18.11)	0.021		
DPAS				0.279		0.279		
NCD			0.66 (0.52–0.84)	0.001	0.66 (0.52–0.84)	0.001	0.70 (0.57–0.87)	0.001
CCL			5.39 (1.35–21.50)	0.017	5.39 (1.35–21.50)	0.017	6.21 (1.73–22.27)	0.005
Logtime, y		0.439		0.835		0.835		0.728

a *OR indicates odds ratio; CI, confidence interval; DPAS, deep perforating artery supply; NCD, nidus-to-CST distance; CCL, the closest CST level; and CST, corticospinal tract*.

b *Not having an OR indicates the variable was excluded from logistic model after stepwise selection*.

**Figure 1:**
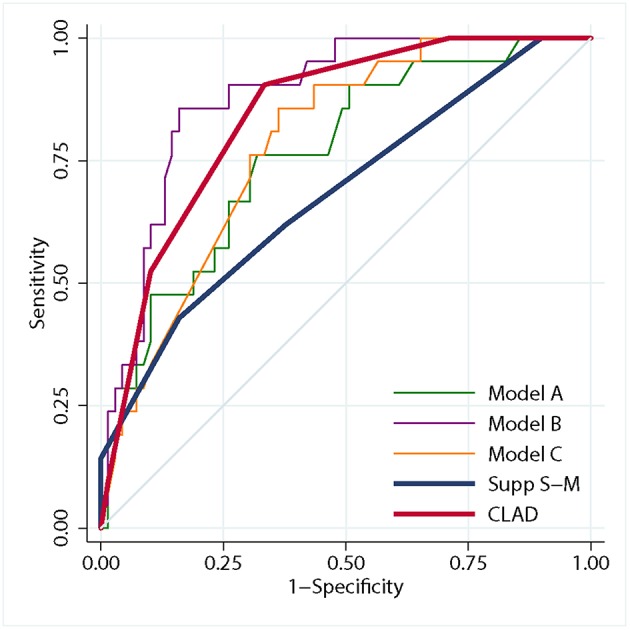
Graph showing receiver operating characteristic analysis for the three logistic models, the CLAD grading scale and the supp Spetzler-Martin grading system. The predictive accuracy of model B (area under purple curve = 0.88) was higher than that of model A (area under green curve = 0.76) and model C (area under orange curve = 0.78). The predictive accuracy of the CLAD grading scale (area under red curve = 0.84) was higher than that of the supp Spetzler-Martin grading system (area under blue curve = 0.68).

The ROC curves of the three generated logistic models are shown in Figure 1. The AUC of model B (0.88, 95% CI 0.81–0.95) was larger than that of model A (0.76, 95% CI 0.65–0.88) and model C (0.78, 95% CI 0.69–0.88). The AUC of the three models were significantly different (*P* < 0.001).

### The Proposed Grading Scale

The new prediction grading scale was constructed on the basis of the logistic model B. According to the β coefficients derived from the model, weight points were attached to patient age, AVM diffuseness, NCD, and CCL (Table 3). These points were summed to obtain the CLAD (closeness, level, age, and diffuseness) grading score (from 1 to 7) of each patient. Patient motor outcome by each supp S-M grade and CLAD grade were shown in Table 4. The risk of persistent postoperative limb weakness rose along with an increasing supp S-M or CLAD grade.

**Table 3:** The point scoring of the proposed CLAD grading scale and the supp S-M grading system^a^.

	**Weight points**	
**Variable**	**CLAD grading**	**Supp S-M grading**
**Size, cm**		
<3		1
3–6		2
> 6		3
**Deep venous drainage**		
No		0
Yes		1
**Eloquence**		
No		0
Yes		1
**Unrupture history**		
No		0
Yes		1
**Age, y**		
<20	0	1
20–40	1	2
> 40	2	3
**Diffuseness**		
Compact	0	0
Diffuse	1	1
**NCD, mm**		
5–10	1	
0–5	2	
0	3	
**CCL**		
Upper	0	
Lower	1	

a *NCD indicates nidus-to-CST distance; CCL, the closest CST level; and CST, corticospinal tract*.

**Table 4:** Patient motor outcome by the supp S-M grade and the CLAD grade.

	**Persistent postop limb weakness**	
**Variable**	**No**	**Yes**
**The supp S-M grade (%)**		
1	0	0
2	0	0
3	7 (100.0)	0
4	9 (81.8)	2 (18.2)
5	27 (81.8)	6 (18.2)
6	15 (78.9)	4 (21.1)
7	11 (64.7)	6 (35.3)
8	0	3 (100.0)
9	0	0
10	0	0
**The CLAD grade (%)**		
1	5 (100)	0
2	15 (100)	0
3	26 (92.9)	2 (7.1)
4	16 (66.7)	8 (33.3)
5	6 (40.0)	9 (60.0)
6	1 (33.3)	2 (66.7)
7	0	0

ROC analysis showed that the AUC of the CLAD grading scale was significantly larger than that of the supp S-M grading system (0.84 [95% CI 0.76–0.92] vs. 0.68 [95% CI 0.55–0.82], *P* = 0.023), which indicated a better predictive accuracy (Figure 1). The 5-fold cross-validation resulted in an AUC of 0.83 similar to 0.84, suggesting that the discrimination of CLAD grading scale was not overly optimistic. The cutoff score was 3.5, which dichotomized the CLAD grades into two groups: a low-risk group (grade 1–3) with a risk of persistent limb weakness of 0–7.1% and a high-risk group (grade 4–7) with a risk of 33.3–66.7%.

## Discussion

AVM resection was an “all-or-nothing” operation, requiring radical excision of the malformed vessels from deep parenchyma. Surrounding brain tissues are liable to be damaged because of the direct dissection or retraction during surgery, as well as the risk of postoperative edema, hemorrhage, or infarction (16). Resection of the nidus close to motor pathways usually carries a high risk of postoperative motor dysfunction. Instead of the hard-to-define eloquence on conventional images, the CST fibers can be reconstructed from motor cortex to medulla oblongata with preoperative DTI data (7). In this series, both NCD, and CCL were evaluated by DTT before AVM resection. In patients with NCD <10 mm, up to 45.6% experienced limb weakness at 7 days after surgery, of whom more than half did not make a full recovery after long-term follow-up. The excessive risk necessitated improving risk prediction and patient selection for AVMs suspected of being close to the CST.

In agreement with our previous research (19), the closeness of the nidus to the CST was found to be a strong independent predictor for motor outcome in the present study. A cutoff point of 5.3 mm was determined by ROC analysis. Considering clinical practicability and possible measuring error, a rounded value of 5 mm was designated as the susceptible distance range around AVM nidus. An NCD of 0 mm meant direct involvement of CST; as the fibers being disrupted or surrounded by malformed vessels, damages seem unavoidable during surgery. An increasing rate of persistent limb weakness of 10.3, 25.0, and 40.7% was respectively, associated with the NCD subgroup of 0, 0–5, and 5–10 mm.

In this series, patients with lower CCL were significantly associated with persistent limb weakness. The corticofugal fibers become compactly arranged at the level of PLIC. Thus, theoretically, a same volume of damage to the lower CST below PLIC can lead to a larger quantity of fiber damages, which would definitely deteriorate motor function. Similar findings were concluded in previous studies of stroke patients. Schiemanck et al. (20) found that the involvement of the CST with a greater density of corticofugal fibers was associated with poor motor recovery after stroke. Puig et al. (10) found that CST damage at the PLIC level was the strongest predictor for unfavorable long-term motor outcome, compared with other CST parts.

Diffuse AVMs have been associated with a higher surgical morbidity in previous studies (21). In agreement with the Lawton-Young grade (17), we identified diffuseness as an independent predictor and incorporated it into the CLAD grading scale. Compact niduses are composed of a tight tangle of vessels with little brain tissue within it, which can be easily dissected with minimal necessary tissue injury. By contrast, the resection of diffuse niduses is frequently challenging because of the vague borders and interspersed white matter. This morphology will inevitably cause sacrifice of the intermixed parenchyma, and can force the dissection plane far from the nidus, leading to excessive tissue injury (21). However, because the quantification of diffuseness remains difficult, the qualitative determination of diffuseness can vary between observers, which seems somewhat problematic. Previously, Spears et al. tested the interobserver variability in determining diffuseness, and found a favorable agreement between experienced neurosurgeons (κ = 0.67) (22).

We found age was another independent predictor for patient long-term motor outcome. Superior outcomes were previously reported in the younger patient group after AVM resection (17, 23). Older age was correlated with a higher risk of persistent limb weakness, which can be partly attributed to worse functional recovery due to the decreased ability of neural regeneration and plasticity. Among the patients with postoperative limb weakness in this series, the patients who finally made a full recovery were significantly younger than those who did not (20.3 ± 2.4 vs. 34.1 ± 3.0, *P* = 0.001). The higher incidence of medical comorbidities and decreased tolerance for surgery in older ages might additionally contribute to this result. Coinciding with Lawton et al. (17) age categories were divided by 20 and 40 years for constructing the CLAD grading scale.

In this study, we found no significant correlation between AVM size and motor outcome. Postoperative motor dysfunction is present only when critical motor regions are damaged, suggesting that a large nidus does not necessarily predict motor dysfunction, but that the AVM location can be predictive by contrast. It has been demonstrated that deep venous drainage and DPAS complicate AVM resection, frequently contributing to increased surgical risks (16, 21). However, deep venous drainage was not associated with poor motor outcome in this series. We attributed this non-significance to the study purpose and the selected population in our study. DPAS showed significance in univariable analysis, but not in the multivariable analysis. Because DPAS usually feeds the deeply located diffuse part of the AVM nidus, we consider that the effect of DPAS might be indirectly addressed by diffuseness in the CLAD grading scale. Previously, rupture history was found to be a protective factor for functional outcome after AVM resection (24), but the assumed protection provided by gliosis or hematoma from bleeding was not confirmed in the present study. We found that gliosis or hematoma was frequently not located between the nidus and CST due to the varying sites and amounts of bleeding, which might compromise the protection of motor function.

**Figure 2:**
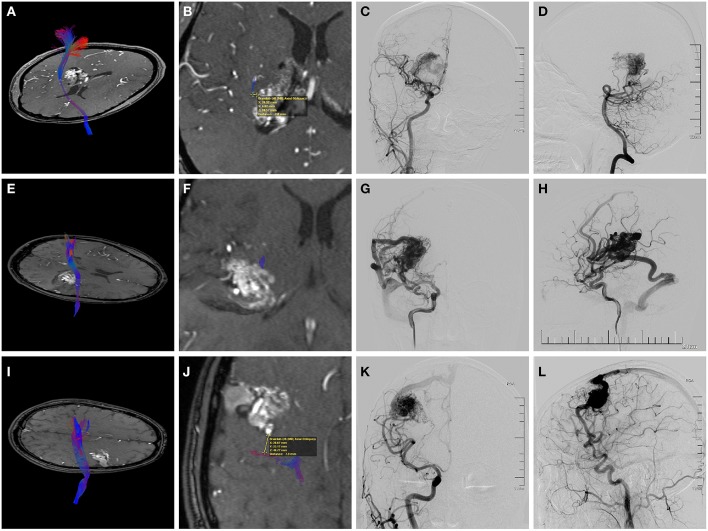
Preoperative magnetic resonance angiography (MRA) fused with the reconstructed corticospinal tract (CST) and digital subtraction angiography (DSA) of three individual AVM patients. **(A–D)** A 38-year-old woman who suffered twice intraventricular hemorrhage before treatment. **(A,B)** Axial MRA showing the deeply located periventricular AVM with the internal capsule involved. Fusion images demonstrated that the shortest nidus-to-CST distance (NCD) was 2.0 mm, and the closest CST level (CCL) was the posterior limb of the internal capsule (PLIC). **(C,D)** Angiograms (anteroposterior view of right ICA injection and lateral view of right VA injection) showing a diffuse nidus with deep venous drainage. Therefore, this patient had a supp Spetzler-Martin (supp S-M) grade of 7 (S2V1E1A2U0D1) and a CLAD grade of 5 (C2L1A1D1). After surgery, she experienced persistent left limb weakness (motor strength of 3-4 at the last follow-up). **(E–H)** A 21-year-old man who presented with remote temporal hemorrhage. **(E,F)** Axial MRA showing the AVM located in the deep sylvian fissure with involvement of the PLIC. The NCD of 0 mm and the CCL of PLIC was demonstrated by image fusion. **(G,H)** Angiograms (right ICA injection; anteroposterior view and lateral view) showing a compact nidus with superficial venous drainage. Therefore, he had a supp S-M grade of 5 (S2V0E1A2U0D0) and a CLAD grade of 5 (C3L1A1D0). He experienced persistent postoperative left upper limb weakness (motor strength of 4). The CLAD grade was more predictive for his motor outcome. **(I–L)** A 29-year-old man who presented with seizures. **(I,J)** Axial MRA showing the unruptured AVM abutting the precentral gyrus. Fusion images showed that the NCD was 7.9 mm, and the CCL was cortex or centrum semiovale. **(K,L)** Angiograms (right ICA injection; anteroposterior view and lateral view) demonstrating superficial venous drainage and a diffuse border (deeply). Therefore, the supp S-M grade was 7 (S2V0E1A2U1D1) and the CLAD grade was 3 (C1L0A1D1). This patient recovered fully postoperatively. The CLAD grade predicted better.

According to our experience as well as past studies (25), local FA close to AVM nidus is lower than that in the contralateral hemisphere in some patients, which can be ascribed to perinidal white matter changes, i.e., edema or gliosis. These perinidal hyperintensities possibly lead to false-negative results, if the same anisotropy threshold is used in fiber tracking (12, 26). Another obstacle compromising the accuracy of CST reconstruction close to the nidus is the susceptibility artifact associated with EPI. These artifacts typically encountered in the vicinity of hemorrhagic nidus may cause geometric and intensity distortions (25). Notably, the GRAPPA technique we used in data scanning has greatly reduced these distortions. Although the clinical use of DTT has been somewhat overshadowed by its inherent technical limitations (27), our findings further evidenced its reasonable reliability in preoperative evaluation, enabling neurosurgeons to determine the spatial relationships between AVMs and motor pathways. We found that introducing tractography-based predictors into the prediction model greatly improved the accuracy of prognosis regarding the selected population. Focusing on motor outcome predicting, the discrimination of the proposed CLAD grading scale was greatly improved compared with that of the supp S-M grading system. Kim et al. validated the predictive accuracy of the supp S-M grading system, and suggested a supp S-M grade ≤ 6 as the boundary for AVM operability (28). The use of CLAD grade can not only further confirm but also can influence the decision-making for AVM patients, in addition to the supp S-M grade (Figure 2). We suggest that the CLAD would be an effective supplement in surgical decision-making.

## Limitation

This study has several limitations. First, technical obstacles associated with DTI and fiber tracking might affect the results of the study. Second, other postoperative complications such as linguistic or visual dysfunction were not analyzed because of the study purpose. Patient selection must require a comprehensive consideration of every possible adverse event. However, we believe that the susceptibility and subsequent influence can vary between different eloquent structures of each subtype function (29); thus, we anticipate that a new prediction method specially focusing on other dysfunctions will be developed. Third, we consistently used anatomical ROIs, and found a good predictive accuracy of CST tractography. It was previously reported that reorganization of the CST was observed in an AVM patient by using functional magnetoencephalography ROI (30). Although this finding has not been further evidenced, attention should be paid to possible CST reorganization when handling individual patients. Last, this single-institution study may be subject to selection bias and confounding. Further validation with more patients from different institutions is needed.

## Conclusions

Shorter NCD and lower CCL were significantly associated with persistent postoperative limb weakness after surgery of AVMs close to the CST. The CLAD grading scale, incorporating NCD, CCL, patient age, and AVM diffuseness, had a high predictive accuracy for long-term motor outcome. We propose the CLAD grading scale as an effective tool to improve preoperative risk prediction and patient selection.

## Data Availability

The datasets generated for this study are available on request to the corresponding author.

## Ethics Statement

The protocol was approved by the institutional review board of Beijing Tiantan Hospital, Capital Medical University. All subjects gave written informed consent in accordance with the Declaration of Helsinki.

## Author Contributions

SW was in charge of supervising the whole study. ML and JW contributed to the conception or design of the work. ML and PJ were responsible for drafting and revising. RG, QL, and SY contributed to data collection. ML and YC were responsible for analysis and interpretation of data.

### Conflict of Interest Statement

The authors declare that the research was conducted in the absence of any commercial or financial relationships that could be construed as a potential conflict of interest.

## Abbreviations

CLAD: a grading system including NCD (closeness)
CCL: patient age and AVM diffuseness
CCL: the closest CST level
DPAS: deep perforating artery supply
NCD: nidus-to-CST distance.
